# Less Data Same Information for Event-Based Sensors: A Bioinspired Filtering and Data Reduction Algorithm

**DOI:** 10.3390/s18124122

**Published:** 2018-11-24

**Authors:** Juan Barrios-Avilés, Alfredo Rosado-Muñoz, Leandro D. Medus, Manuel Bataller-Mompeán, Juan F. Guerrero-Martínez

**Affiliations:** Group for Digital Design and Processing, Department of Electronic Engineering, School of Engineering, Universitat de Valencia, Burjassot, 46100 Valencia, Spain; juan.barrios@uv.es (J.B.-A.); leandro.medus@ext.uv.es (L.D.M.); Manuel.Bataller@uv.es (M.B.-M.); juan.guerrero@uv.es (J.F.G.-M.)

**Keywords:** neuromorphic systems, event-based sensors, dynamic vision sensor, bioinspired event filtering, FPGA implementation, spike-based, event data reduction

## Abstract

Sensors provide data which need to be processed after acquisition to remove noise and extract relevant information. When the sensor is a network node and acquired data are to be transmitted to other nodes (e.g., through Ethernet), the amount of generated data from multiple nodes can overload the communication channel. The reduction of generated data implies the possibility of lower hardware requirements and less power consumption for the hardware devices. This work proposes a filtering algorithm (LDSI—Less Data Same Information) which reduces the generated data from event-based sensors without loss of relevant information. It is a bioinspired filter, i.e., event data are processed using a structure resembling biological neuronal information processing. The filter is fully configurable, from a “transparent mode” to a very restrictive mode. Based on an analysis of configuration parameters, three main configurations are given: weak, medium and restrictive. Using data from a DVS event camera, results for a similarity detection algorithm show that event data can be reduced up to 30% while maintaining the same similarity index when compared to unfiltered data. Data reduction can reach 85% with a penalty of 15% in similarity index compared to the original data. An object tracking algorithm was also used to compare results of the proposed filter with other existing filter. The LDSI filter provides less error (4.86 ± 1.87) when compared to the background activity filter (5.01 ± 1.93). The algorithm was tested under a PC using pre-recorded datasets, and its FPGA implementation was also carried out. A Xilinx Virtex6 FPGA received data from a 128 × 128 DVS camera, applied the LDSI algorithm, created a AER dataflow and sent the data to the PC for data analysis and visualization. The FPGA could run at 177 MHz clock speed with a low resource usage (671 LUT and 40 Block RAM for the whole system), showing real time operation capabilities and very low resource usage. The results show that, using an adequate filter parameter tuning, the relevant information from the scene is kept while fewer events are generated (i.e., fewer generated data).

## 1. Introduction

The development of event-based sensors is an important topic. Vision sensors are common [1,2] but other event-based sensors exist, especially in those areas where bioinspired sensors are developed, e.g., artificial cochleas [3] and olfactory systems [4,5]. On the one hand, the data received from the sensors consist on events (also called spikes) which greatly differ from the traditional data values received form sensors (typically, analog values). For this reason, further data processing requires special algorithms and techniques. On the other hand, new devices (network nodes) are constantly added into a laboratory or industrial communication network, increasing the volume of data transmitted. Nowadays, data transfer is increasing at a higher pace than the supported bandwidth due to the addition of advanced equipment generating and transmitting many data and causing the saturation of communication networks [6]. This is a problem, especially in those applications where real-time and low-latency are required [7]. In the case of vision sensors, which generate many data, event-based encoding techniques can be a solution so that vision sensors can be connected into an existing communication network. In frame-based cameras, it is common to use a separated ethernet network for image transmission. Event-based cameras produce data in the form of events, asynchronously [8]. Data are generated only when there is a difference in light intensity received by any of the sensors (pixels) arranged in an array. Each pixel of the camera that can sense this difference in intensity will produce an event if such difference is bigger than a threshold setting that can be adjusted. The generated event includes information about the address of the pixel in the sensor where the threshold was exceeded, together with a time stamp in order to generate a unique event, not just in space but also in time. Typically, positive or negative events are generated if the event is caused by an intensity increment or decrement, respectively. These changes in intensity are mainly caused by changes in the visual scene, which generates event data related to the scene. This behaviour is similar to a mammal brain [9], which leads to use neuromorphic systems [10] for further information processing [11], feature extraction, scene detection [12] and filtering [13,14].

Proper lighting is a key factor in traditional industrial vision systems since it is difficult to maintain a constant light due to a constantly changing environment. Traditional solutions require the use of specific lighting systems suited for specific applications [15,16,17]. Event-based cameras minimize light effects since only pixel intensity differences are considered and no specific light intensity is required, independently of light conditions.

Currently, applications working with event-based cameras have been mainly developed with research purposes, emulating a neuromorphic system [18,19,20]. However, only a few deal with the data transfer of event data [21,22]. Event-based systems have not yet achieved the desirable spread in industrial environments to benefit from their advantages. However, current event-based systems still use a high bandwidth to transmit data, higher than a typical industrial communication system could handle. According to Farabet et al. [23], an advanced event-based sensor with about 1 million neurons might generate up to 10^8^ million events per second; in a relatively simple example, the authors showed an experimental test where 8 million events per second are generated. This number of events can make an event-based system require a similar bandwidth to be transmitted to frame-based vision, making their advantages overshadowed and conventional machine vision systems (frame-based) being still used in industry environments. Nowadays, event-based processing techniques are focused in producing better data for pattern recognition in neuromorphic systems [24,25] and machine learning [26] rather than event data pre-processing which could ease the task of data transmission and further machine learning or other classification, prediction or recognition algorithms due to more clear data. In [27], a filtering algorithm is proposed, aiming a similar goal to our proposed work. However, its complexity (based on two-layer processing with neural network processing) requires a high computational cost, not being feasible for on-chip implementation.

The main aim of this work was to design an algorithm able to filter data obtained from event-based sensors, generating fewer events while keeping relevant information from the scene. Thus, the volume of transmitted data from the sensor can be reduced, requiring less bandwidth, lower energy consumption and less storage, which are very important issues for data transmission in communication networks and data storage. Additionally, not only reduced data transfer is required but also real-time response needs to be provided, which means that a low complexity, yet effective, algorithm must be developed. Some works are focused on developing and improving systems for data exchange between two or more bioinspired devices [21,22], transmitting original sensor data to the processing unit, i.e., a neuromorphic system typically composed of spiking neurons.

Taking the above into consideration, an algorithm was designed and tested for processing and filtering data from event-based sensors. For this reason, we call it “Less Data Same Information (LDSI)”. Since it is valid for different event-based sensors, we focused on event-based cameras. This technique is based on how biological neurons work, i.e., acquired data consist of on–off spike sequences. This algorithm is fully configurable, with the main goal of providing adjustable results of filtering and data reduction depending on the final application. The use of this filter reduces the volume of data received by a neuromorphic system for classification, prediction, or any other application. Several of the factors inherent to industrial vision systems are considered: events generated by unit of time, noise, size of the image, and strong light changes, among others.

The used materials and existing techniques are detailed in Section 2. Section 3 details the proposed LDSI bioinspired algorithm, with results provided in Section 4, including real-time performance with FPGA implementation. Finally, Section 5 and Section 6 discuss the results and provide conclusions, respectively.

## 2. Materials and Methods

**Figure 1:**
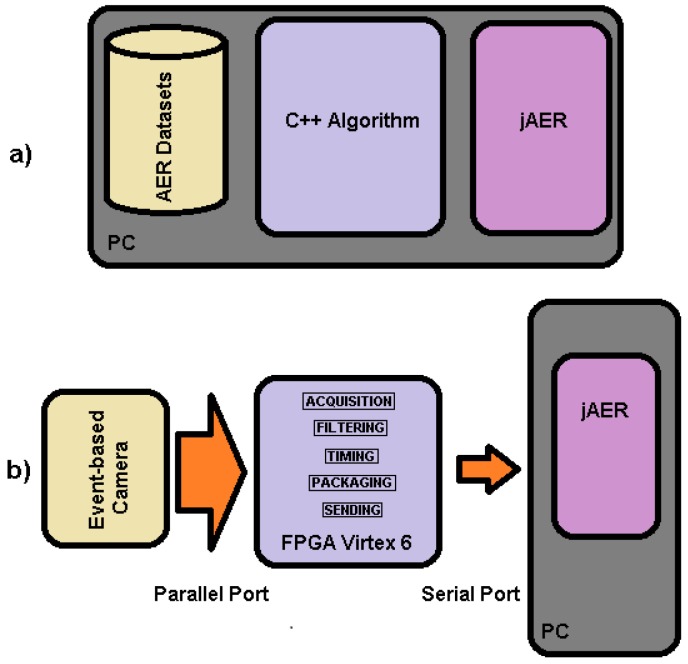
Procedures used to develop and test the LDSI algorithm: (**a**) offline configuration, where, from left to right, AER dataset recorded with event-based cameras was read and applied to the PC programmed LDSI algorithm whose results were provided to jAER for visualization; and (**b**) online testing, where an event-based camera was connected through its parallel port to an FPGA where the LDSI algorithm was computed and output data were properly encapsulated and sent to a PC for visualization and data-logging through a serial port.

Common standard platforms and tools in the event-processing field are used. The proposed algorithm must be compatible with a wide range of existing devices, both for event-based sensors as the input, and event-based processing units. Under this guideline, Address Event Representation (AER) for event transmission was used [28]. For data visualization, jAER software was used [29]. Figure 1 shows the two main approaches for development and testing of the algorithm. Initially, the algorithm was tested offline for event-based data from a database. It was developed in C++ language and performance evaluation and visualization was directly done in jAER (Figure 1a). Once the algorithm was developed, real-time performance was verified in an online real environment using an event-based camera connected to an FPGA where the algorithm was implemented and the results were transmitted to a PC in AER format, for final jAER visualization (Figure 1b). Specifically, the implementation was done in a Virtex-6 XC6VLX240T-1FFG1156 FPGA used in the ML605 evaluation board by Xilinx. The camera was a Dynamic Vision System (DVS) from Inilabs [2] connected to the FPGA through its parallel port [30]. The parallel port is a 15-line AER bus: 7-lines for Y-axis address, next 7-lines for the X-axis address of the active pixel, and one line for the polarity. The read was controlled by two extra lines (“REQ” and “ACK”) for transaction control. In addition, the FPGA was connected through a serial port to a PC; the connection, baud-rate (921,600 bps) and data protocol were made to be compatible with jAER software, where the result of the algorithm was verified.

Address-Event Representation is an efficient and universal method of transmitting event data. It was proposed by Sivilotti in 1991 [31] and, since then, it has been widely adopted in the neuromorphic hardware field. With this type of encoding, each device has its own event space defined and it transmits information only in the case of state changes in any of the sensor receptors (pixels, in the case of an event-based camera). As an example, for a silicon retina, the event space will be the whole pixel matrix, where every pixel is an independent event source. Upon a threshold event in a pixel, the information about the change is encoded into a numerical value, typically, XY coordinates of the changing pixel. Thus, as only significant changes generate new data, the amount of information that the retina generates is several orders of magnitude lower when compared to a frame-based vision camera where all pixel values are transmitted every new frame regardless of pixel intensity change, generating redundant data. Using AER, the areas of interest (areas where the image has changed) is automatically identified since only data from this area are generated. For instance, in a ball intercept task, the average event stream is 20 kEvts/s corresponding to a 40 kB/s streaming speed. Using a frame-based camera with the same time resolution would require 6.6 MB/s of raw data stream [32]. The AER communication is suitable for low latency systems. In theory, all event sources are completely independent and asynchronous, and generate an AER data packet immediately after receiving the event. In practical applications, simultaneous event collisions are common and the event source usually includes an event management and scheduling algorithm to prevent data loss.

Nowadays, there exist several protocols to encapsulate AER data. However, two main consolidated protocols are commonly used: AER1.0 and AER2.0 [26]. Currently, a new protocol version (AER3.0) is being tested for more complex and flexible data transfer between event-based devices [33]. In this application, we used AER1.0 for a dvs128 format, compatible with jAER. AER1.0 requires fewer bytes for the frame construction and, therefore, less transmission time. The protocol frame was built as follows:

1. The first bit of the first byte is used to align the data (always “1”) at reception.
2. The following seven bits are the Y-axis coordinate of the pixel location.
3. The first bit of the second byte represents the polarity of the event, increment or decrement of the measured magnitude.
4. The next seven bits represent the X-axis coordinate of the pixel location.
5. Finally, the next four bytes contain the time elapsed (µs) since the last event generated (timestamp).

The operating protocol used in this case was the same as for an “edvs128” camera [34]. Proper data encapsulation was performed by the FPGA in the case of online operation.

## 3. Less Data Same Information (LDSI) Algorithm with Event-Based Encoding

**Figure 2:**
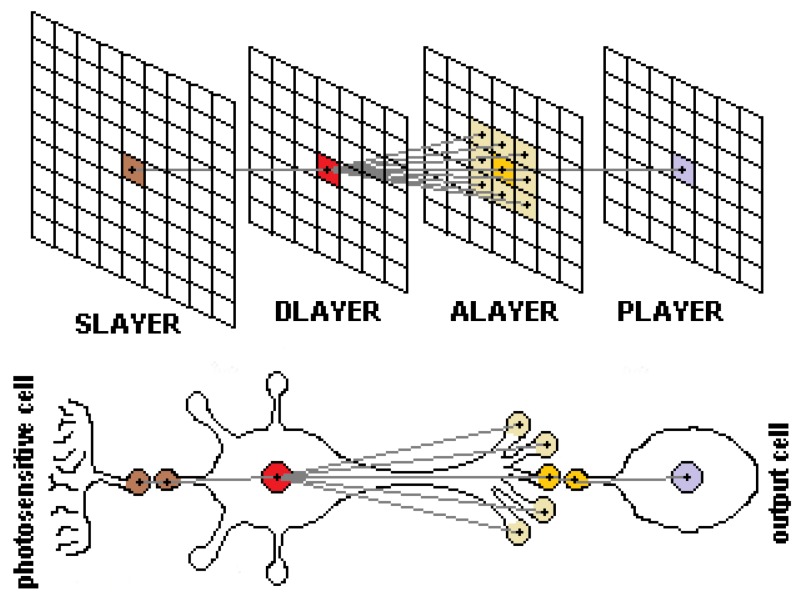
(**Top**) Interrelation between layers created in the LDSI algorithm. Lines between layers show the interconnections and data flow from the events generated in the input sensory layer (**Slayer**) corresponding to an event-based sensor (DVS camera in this case) to the output layer **Player** with filtered data. (**Bottom**) Equivalence of the proposed layer model in the LDSI algorithm with a biological neuron.

This work proposed a novel algorithm, not only filtering noise generated in event-based cameras, but also reducing the number of redundant or irrelevant data. The proposed LDSI algorithm has a neuromorphic basis since it is based on spiking cells similar to those described by Izhikevich [9]. Specifically, it was inspired by the bipolar cells of the retina. However, the goal of this work was not to emulate a neuromorphic system but take advantage of some biological neurons concepts to reduce data transmission without loss of information. The defined model and its comparison to a biological neuron are shown in Figure 2. The layer-based model for event processing can be associated with sensory units in the sensory layer **Slayer** (pixels in case of a camera) which act as the dendrites feeding data to the nucleus (**Dlayer**) also forwarding information to synaptic terminals (**Alayer**). Each synaptic terminal in **Alayer** produces a final output represented in the output layer **Player** which can be considered as the next **Dlayer** in a successive chain of neurons.

Thus, the model defines a single neuron composed of two units associated with the nucleus (**Dlayer**) and the axon or synaptic terminals **Alayer**, being M×N units in size. These units are arranged in two layers forming a neuronal-like structure. Each layer is defined by a bidimensional matrix of units identified by its xy coordinates in the matrix (Figure 2). Each unit in **Dlayer** and **Alayer** receive events from the input layer **Slayer** ((M+2)×(N+2) matrix size) and modifies their internal potential values, similar to biological neurons. A unit $D_{xy}$ in **Dlayer** receives input events from the same xy position in the event generation layer (e.g., a sensory layer in an event sensor, or the output of a preceding layer). Then, the unit modifies its internal potential $\vartheta_{D}\left(x,y\right)$, which can be associated to the potential of the nucleus in a biological cell. Simultaneously, the units in **Alayer** modify its internal potential $\vartheta_{A}\left(x,y\right)$ due to input events received in **Dlayer** units located in xy, and the vicinity. Each unit in a layer modifies its internal potential and, when potential in both **Dlayer** and **Alayer** is above a threshold, the unit in **Alayer** generates an output event, reflected in **Player**, which has the same structure and size as the input layer ((M+2)×(N+2)). This approach allows this LDSI filter to be included between already existing event processing modules since the **Player** output can be interpreted as the original input layer. This approach is the same as in other processing areas where different filters may be added as pre-processing.

**Figure 3:**
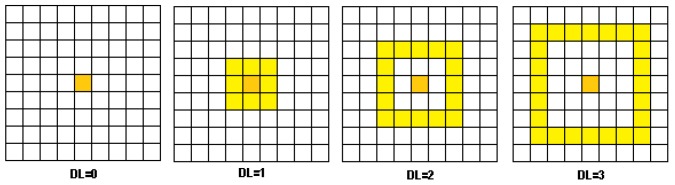
Interconnections existing between **Dlayer** and **Alayer** according to the DL parameter value.

The LDSI filter can define the number of neighbour units from **Dlayer** affecting a unit $A_{xy}$ in **Alayer**, which is defined by the Depthlevel,DL∈N parameter (Figure 3). This effect resembles a receptive field affecting potential in units nearby the generation of an event.

In addition to **DL** already explained, the following parameters related to the units in the layers are defined:

- **Excitation level in Dlayer (ELD)**: Magnitude of the potential that a unit in the xy unit of **Dlayer** increases when an event is received from the unit located in the same xy unit in **Slayer**.
- **Excitation level in Alayer (ELA)**: The potential increment in the xy unit of **Alayer** due to an event in the same xy unit of **Dlayer**.
- **Excitation level in Alayer neighbouring units (ELAN)**: When an event is produced in an xy unit of **Dlayer**, ELAN corresponds to the potential increment of units in **Alayer** the vicinity of the xy unit. The number of affected neighbour units varies according to the **DL** value.
- **Threshold potential level in Dlayer (TPD)**: Defines the minimum value of excitation required for a certain unit in **Dlayer** to generate an output event.
- **Threshold potential level in Alayer (TPA)**: Defines the minimum value of excitation required for a certain unit in **Alayer** to generate an output event.
- **Decrement of potential in Dlayer (DPD)**: The value of potential to be decremented in **Dlayer** once MTR has elapsed.
- **Decrement of potential in Alayer (DPA)**: The value of potential to be decremented in **Alayer** once MTR has elapsed.

For event-based systems, delay between events affect how potential in a unit changes. The following parameters concerning time delay of events are defined:

- **Actualtimestamp (AT)**: The timestamp of the actual event present in a certain connection.
- **Lasttimestamp (LT)**: The timestamp of the previous event received in a certain connection.
- **Deltatime (DT)**: Time difference between the actual and the previous event coming from a certain connection. If this value is higher than MTR, the potential in the unit is decreased.
- **Maximum time to remember (MTR)**: Defines the maximum time between two events that the potential value in unit from layers **Dlayer** and **Alayer** can remain before being degraded. This parameter can be associated to a forgetting factor in the unit.

Upon an input event in the xy position of **Slayer**, the potential in the xy unit in **Dlayer** and the xy and **DL** neighbouring units in **Alayer** is increased by its corresponding excitation value. Equation (1) shows how potential $\vartheta_{D}\left(x,y\right)$ in an xy unit of **Dlayer** changes and Equation (2) shows the calculated potential $\vartheta_{A}\left(x,y\right)$ in an xy unit in **Alayer**, as a function of the above defined parameters. It is important to note that, for the same input events, potential in each layer evolves in a different form. In case of **Dlayer**, Equation (1) gives the mathematical description, and Equation (2) in the case of **Alayer**.

In case of **Dlayer**, potential is increased only when the event is received by exactly the same xy unit in **Slayer**; for **Alayer**, the potential is increased when an event exist in the xy unit in **Slayer**, or a **DL** neighbouring position. Potential is decreased if no event is received after a certain time defined by MTR, with zero limit, i.e., potential cannot be negative. When conditions are met, an output event is generated and, immediately, potential goes to zero until new events arrive. To generate an output spike in a xy unit in **Alayer**, it is important to note that this two-layer model requires that both potential in the xy unit from **Dlayer** and xy unit from **Alayer** are above TPD and TPA thresholds, respectively.

(1) $$\vartheta_{D}{\left(x,y\right)}_{t+1}=\left\{\begin{array}{c} \vartheta_{D}{\left(x,y\right)}_{t}+ELD,\;if\;event\;in\;D\left(x,y\right)\\ \vartheta_{D}{\left(x,y\right)}_{t}-DPD,\;\;DT\geq MTR\\ 0,\;\;\;\;(\vartheta_{D}{\left(x,y\right)}_{t}\geq TPD)\\ \;\;\;\;\;AND\;(\vartheta_{A}{\left(x,y\right)}_{t}\geq TPA)\\ 0,\;\;\;\;(\vartheta_{D}{\left(x,y\right)}_{t}-DPD)\leq 0\\ \vartheta_{D}{\left(x,y\right)}_{t},\;no\;event\;AND\;DT<MTR \end{array}\right.$$

(2) $$\vartheta_{A}{\left(x,y\right)}_{t+1}=\left\{\begin{array}{c} \vartheta_{A}{\left(x,y\right)}_{t}+ELA,\;if\;event\;in\;D\left(x,y\right)\\ \vartheta_{A}{\left(x,y\right)}_{t}+ELAN,\;if\;event\;in\;DL\;\\ \;\;\;\;\;\;\;\;vicinity\;of\;D(x,y)\\ \;\;\;\;\;\;\;\;AND\;DT<MTR\\ \vartheta_{A}{\left(x,y\right)}_{t}-DPA,\;\;DT\geq MTR\\ 1,\;\;\;\;(\vartheta_{D}{\left(x,y\right)}_{t}\geq TPD)\\ \;\;\;\;\;AND\;(\vartheta_{A}{\left(x,y\right)}_{t}\geq TPA)\\ 0,\;\;\;\;\vartheta_{A}{\left(x,y\right)}_{t}=1\\ 0,\;\;\;\;(\vartheta_{A}{\left(x,y\right)}_{t}-DPA)\leq 0\\ \vartheta_{A}{\left(x,y\right)}_{t},\;no\;event\;AND\;DT<MTR \end{array}\right.$$

**Figure 4:**
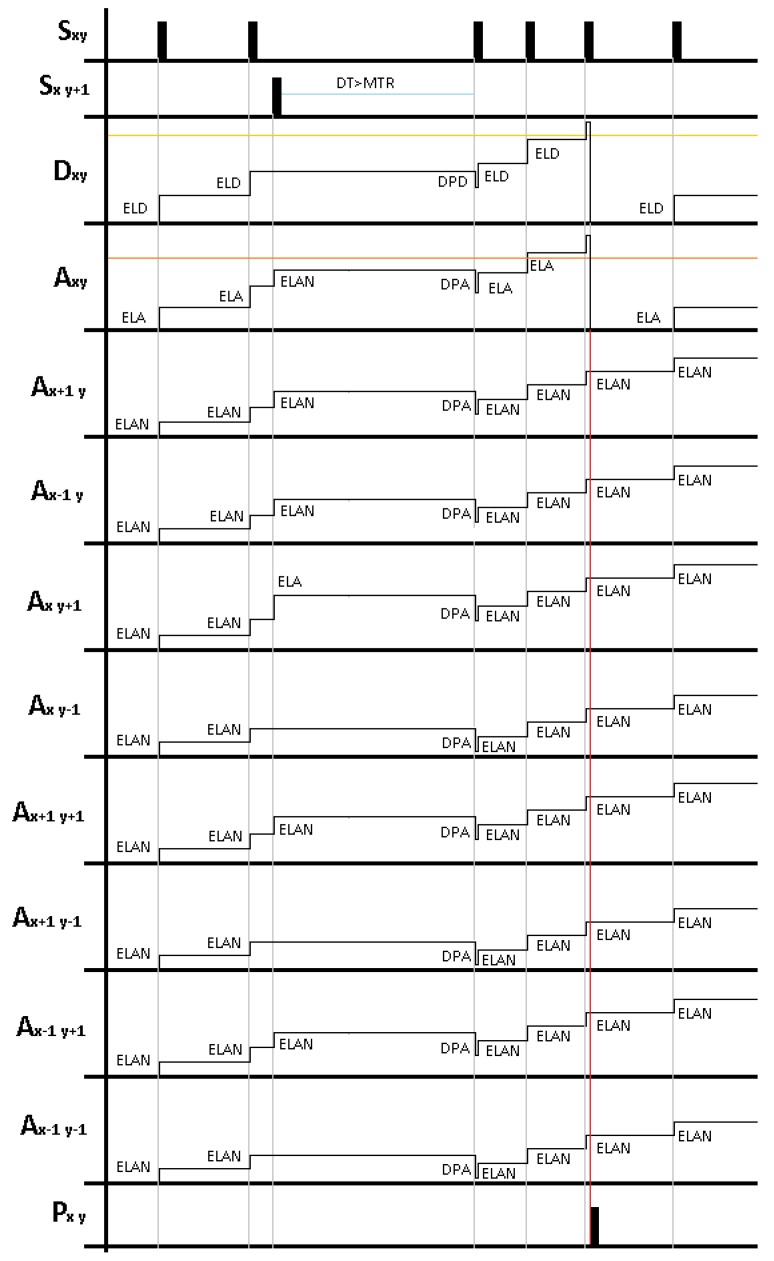
Event processing example in case of input events in two units of **Slayer**, xy and x(y+1). The potential value in each unit increases with input events and, when above a threshold, an output event is generated. If no events exist during a time defined by “MTR”, the potential is decreased. In the case of **Dlayer** layer, only received events from the same xy unit in the previous layer increase its potential. For **Alayer**, events received from neighbour units also increase the potential. In each case, a different potential value can be defined. An output event (valued “1”) is generated when the xy units in **Dlayer** and **Alayer** are above their respective threshold, simultaneously.

Figure 4 shows an example for the behaviour of the LDSI algorithm. In this case, **Slayer** units located in xy and x(y+1) are generating events, which impact in the potential of several units in **Dlayer** and **Alayer**. According to the behaviour described in Equations (1) and (2), the figure shows the potential evolution for an xy unit in **Dlayer**, and the xy unit and all surrounding units in **Alayer**, assuming DL=1. It is important to note that units in **Dlayer** are only affected by events in the same xy position while units in **Alayer** are affected by events in the xy unit and surrounding positions, with different potential increase depending on the event location. Additionally, for each new incoming event in **Slayer**, the time difference between the current time and the time of last event is evaluated; potential for all units in **Dlayer** and **Alayer** is decreased in the case this inter-event time is higher than MTR. When the potential of the xy units in both **Dlayer** and **Alayer** is above their respective threshold TPD and TPA, an output event is generated in the xy unit of **Player**.

The proposed model in this algorithm has two main characteristics: first, those events distant in time have a negative impact on output event generation since they decrease the potential; and, second, those spatially distant events do not contribute to potential increase in units located far from a **DL** distance. These facts allow discarding and providing low consideration to unexpected events as spurious noise events. This event processing model resembles how the mammalian brain continuously receives many data in the form of events, and, depending on the connections of the neurons and their excitation levels (strength), an output event is generated. Algorithm 1 shows how each input event from **Slayer** is processed in the subsequent layers.

### Test Methodology

All parameters are integer values. Experimentally, we determined that the range of ELD, ELA, ELAN, TPD, TPA, DPD and DPA parameters should be kept between 0 and 10. Otherwise, a high computational cost is required without extra benefits. The MTR parameter is given as time units. Depending on the given values to all the parameters, the results can be adjusted to different levels of filtering. Despite the multiple possibilities and parameter value combinations, three main parameter sets were defined to provide weak, medium or restrictive level of filtering.

The LDSI algorithm was developed in two stages. First, we tested the algorithm in an “offline” environment, programming the algorithm in C++ language with the purpose of analysing its behaviour when applying different AER data from various scenes already pre-recorded as data files. The second stage was the LDSI “online” implementation where the algorithm was embedded in an FPGA so that the device obtained data from a real event-based camera, applied the LDSI algorithm and sent the resulting data in the proper format for jAER PC software reading and visualization.

For the algorithm development in the “offline” environment, we used a pre-recorded dataset publicly available on websites from other groups working with AER data processing [29,35]. The algorithm was applied with different datasets, each showing a different scene, and all of them with different noise levels, sizes and quantities of event data per unit of time. The goal of this test was to analyse the algorithm performance under different conditions and obtain enough data under different input event conditions to analyse parameter interrelations obtaining different output results.

For the “online” LDSI algorithm implementation in hardware, the algorithm was migrated from C++ to VHDL so that a more optimized computation was obtained in terms of parallelization, speed of operation and logic resource usage. It was tested in a Virtex 6 FPGA to verify the performance and compare results with the “offline” algorithm by connecting the FPGA with jAER software. The results of the “online” and “offline” LDSI show that the output events generated with the algorithm implemented in the FPGA were correct and coherent with those generated by the PC software implementation.

**Algorithm 1** LDSI algorithm: Computation is performed when events exist in **Slayer**. Output events in the filter are only generated if potential is above threshold.**Require:** Event-based inputs from the sensory layer **Slayer** **Ensure:** Events addresses inside **Slayer**
  1:**if**$Slayer_{INPUT\_EVENT}$ in (x,y) **then**  2: {– Potential increase}  3: DT=AT−LT  4: $D_{\left(x,y\right)}=D_{\left(x,y\right)}+ELD$  5: $A_{\left(x,y\right)}=A_{\left(x,y\right)}+ELA$  6: **for** i=−DLtoDL **do**  7:  **for** j=−DLtoDL **do**  8:   $A_{\left(x+i,y+j\right)}=A_{\left(x+i,y+j\right)}+ELAN$  9:  **end for** 10: **end for** 11: **if** DT<MTR **then** 12:  {– Potential decrease} 13:  **for all** i=0to((M−2)∗DL) **do** 14:   **for all** j=0to((N−2)∗DL) **do** 15:    **if** $D_{\left(i,j\right)}>=DPD$ **then** 16:     $D_{\left(i,j\right)}=D_{\left(i,j\right)}-DPD$ 17:    **else** 18:     $D_{\left(i,j\right)}=0$ 19:    **end if** 20:    **if** $A_{\left(i,j\right)}>=DPA$ **then** 21:     $A_{\left(i,j\right)}=A_{\left(i,j\right)}-DPA$ 22:    **else** 23:     $A_{\left(i,j\right)}=0$ 24:    **end if** 25:   **end for** 26:  **end for** 27: **end if** 28: **if** $D_{\left(x,y\right)}>=TPD$ AND $A_{\left(x,y\right)}>=TPA$ **then** 29:  $P_{\left(x,y\right)}=EVENT$ 30:  $D_{\left(x,y\right)}=0$ 31:  $A_{\left(x,y\right)}=0$ 32: **else** 33:  $P_{\left(x,y\right)}=NULL$ 34: **end if** 35: LT=AT 36:**end if**

## 4. Results

**Figure 5:**
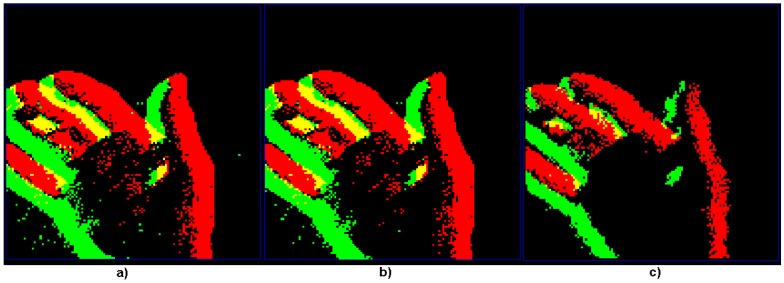
Comparison among the original DVS camera data, jAER “background activity filter” and the proposed LDSI algorithm: (**a**) original events from event-based camera with no filter, where noise and repetitive data were generated, mainly at the object borders; (**b**) result after applying the background noise jAER built-in filter with restrictive parameters; and (**c**) events produced after applying the LDSI algorithm with parameters selected for a compromise between data reduction and loss of main data of the scene (medium filter).

A real scene of a slowly moving hand was captured from a DVS event camera. The same scene is compared when no filtering is applied (Figure 5a), using the existing jAER “background activity filter” (Figure 5b), and the LDSI filter using a medium level of filtering (Figure 5c). This figure shows that the LDSI result not only provided smoother and better edge definition but also reduced the produced data (generated events) compared to the original: 7974 kBytes for the LDSI versus 13,348 kBytes for jAER filter and 15,796 kBytes for the unfiltered.

To obtain a complete evaluation of the LDSI algorithm, 260 sets of parameters were tested, iterating each combination 100,000 times. Output data were analysed through a range of parameter combinations to find an interrelation between the parameters and the filter output data. The results produced by the algorithm varied from a high data reduction level and noise removal, including some removal of data from the area of interest for certain parameter values, to a “transparent mode” where most of the incoming data were transferred to the output. Thus, the parameter configuration allows the filter to be tuned according to the application or user requirements, from a very low to a highly restrictive mode. In any case, the LDSI never generated more output events than the input events and it never blurred or deformed the scene. In some cases, it was possible to obtain zero output events, i.e., null data output from the filter.

**Figure 6:**
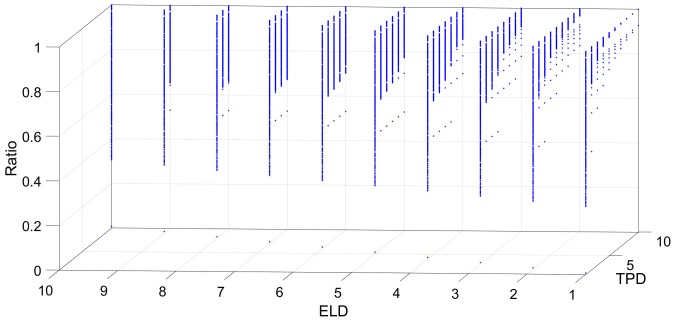
Output events generated by the LDSI algorithm depending on ELD and TPD values. An increase of events is produced when ELD increases. In the left corner of the graph, it is possible to see how low values of ELD at high values of TPD restricts the event production.

**Figure 7:**
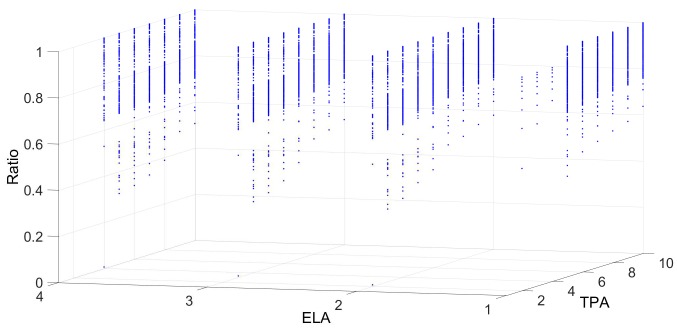
Outputs events as a function of the excitation level (ELA) and threshold (TPA) in **Alayer**. A value of TPA higher than ELA reduces the production of events, thus filtering noise but also some loss of data in the main scene appears.

**Figure 8:**
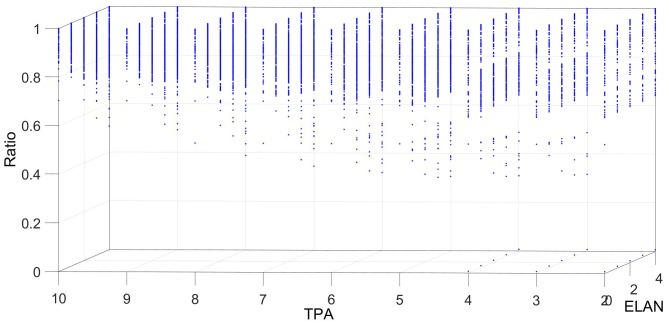
Relationship between the excitation level in neighbours (ELAN) and its associated threshold TPA. The production of output events increases in the case of high TPA and high ELAN.

**Figure 9:**
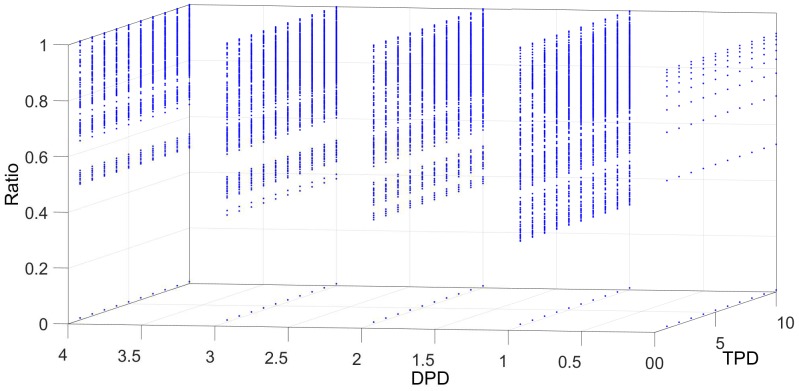
Relationship between the decrement potential level (DPD) and the threshold (TPD) in **Dlayer**. No direct relationship in event reduction among these values is found.

**Figure 10:**
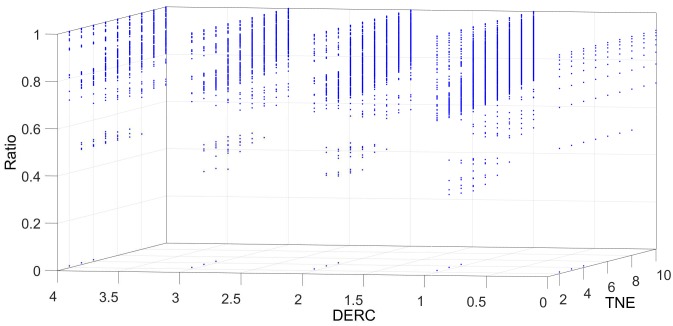
Relationship between the potential level (DPA) and the threshold (TPA) in **Alayer**. Only in case of low DPA or TPA values, the number of output events is reduced.

Figure 6, Figure 7, Figure 8, Figure 9 and Figure 10 show the interrelation between some parameters and how they affect the final result of produced events. To discard non-sense resulting data as zero events at the output, some obvious combinations of parameters were not considered. Some parameters influenced noise removal while others influenced output data production.

Figure 6 shows the number of output events generated by the LDSI, depending on the TPD threshold and ELD values in **Dlayer**, while keeping constant the rest of the parameters. If ELD value is close to TPD value, an output event is more likely to be produced upon input events and then, input and output events will be very similar. For this reason, ELD values higher than TPD were discarded since that combination produces exactly the same data as the original, converting the algorithm into a repeater. On the other hand, if the ELD value is much lower than TPD, the filter will be very restrictive and fewer data will be produced, but the noise will not necessarily be discarded, as it will also discard valid events from the scene.

Figure 7 shows the relation between ELA and TPA (excitation and threshold in **Alayer**). As seen, different variations and combinations of ELA and TPA do not provide a significant modification in the output events. However, the behaviour is greatly affected by TPD and ELD since events arriving to **Alayer** come from **Dlayer** and, thus, only in the case of low values of ELA and TPA, the generated events are reduced.

Regarding the relationship between the threshold TPA and the excitation level of neighbours ELAN, Figure 8 shows that produced events are increased in the case of a high value in both parameters. The result indicates that, as ELAN contributes to the potential, a high value increases the possibility of producing output events since the potential in a certain unit xy increases faster.

Figure 9 shows the relationship between the threshold level TPD and the decrement level in absence of input events (DPD), in **Dlayer**. In this case, we can observe that, for the same TPD value, a reduction in produced events appears when DPD increases. This is a desired effect; it reduces potential if no input events appear. Thus, potential is highly reduced due to high values of DPD and, then, fewer output events are generated.

Concerning the behaviour of **Alayer** in relation to the potential decrease due to the absence of input events (DPD), Figure 10 shows the output events produced depending on TPA and DPA values. In this case, a similar effect with fewer variations than in **Dlayer** appears. A slight decrease in generated data is observed when DPD increases.

Despite the parameters of the filter are fully configurable, after analysing the results, the following conclusions can be obtained:

1. For applications where a high ratio of noise versus the main data is present, the LDSI algorithm has better performance with lower values of MTR.
2. Regardless of the quantity of noise in relation to the relevant data, it is important to define low values of ELA but preferably higher than ELAN, and TPA higher than both ELA and ELAN, which improves the noise removal.
3. When the noise is not a problem and the goal is to obtain a clear distinction of edges from the object in the scene, it is recommended to increase the value of ELD and, at same proportion, DPD and DPA.
4. Finally, it is necessary to avoid configuration parameters where results could be predictable such as ELD and ELA being equal to zero, which will produce zero events. On the contrary, TPD and TPA values lower than ELD and ELA, respectively, will produce the same output data as the input.

These statements are not mathematical facts because they depend on multiple variables such as the ratio between noise and main data of the scene, speed of the objects moving through the scene, hot pixels, size of the sensor, fast change of light (intensity), etc. However, after several tests, it was possible to realize that, under similar conditions, the result of the LDSI algorithm are consistent.

**Figure 11:**
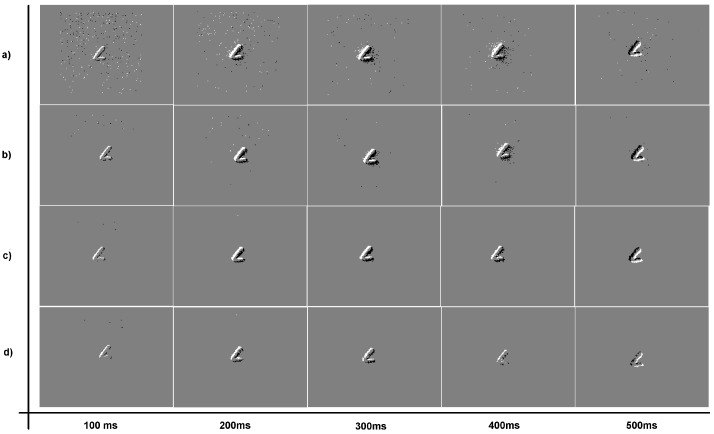
Image sequence of 500 ms (100 ms per image): (**a**) original data without LDSI; (**b**) low data reduction and weak noise removal; (**c**) medium data reduction and medium noise removal; and (**d**) high data reduction with restrictive parameters and high noise removal.

Figure 11 shows the output events under three different LDSI parameterization (weak, medium and restrictive) in comparison with the original input event data, for five different sequences (500 ms each). Five frames of a handwritten letter “L” smoothly moving side to side are displayed. Sequence (a) represents the original input events without filter; Sequence (b) represents the LDSI result with a data reduction of 33% with respect to the original image and parameters adjusted as a weak filter mode; Sequence (c) shows a 50% data reduction with medium filtering options; and Sequence (d) is the resulting sequence produced with a restrictive LDSI parameter configuration, obtaining 85% data reduction. In the case that elimination of noise close to the relevant scene information is required, a more restrictive parameter configuration is recommended, with high values of TPD and TPA with respect to ELD, ELA and ELAN, together with a low MTR value.

Table 1 shows the parameter values corresponding to weak, medium and restrictive data reduction LDSI filter parameter values, together with the number of generated data for each case. A low output data reduction implies weak filtering with low noise removal while a high data reduction means high filtering with the risk of losing relevant features in the scene. In any case, the type of scene greatly affects the results and, thus, parameters must be chosen according to the target scene or features to be extracted.

### FPGA Hardware Implementation

Concerning hardware implementation, the LDSI filter was implemented in a Xilinx Virtex6 6vlx240tlff1156-1l device. The implemented system included serial port connectivity to a PC for jAER data exchange so that pre-recorded events could be sent to the FPGA for LDSI filtering and then returned back to the PC for jAER analysis and visualization. Additionally, a DVS camera can be connected to the FPGA for real-time data event input. The FGPA applies the LDSI algorithm and filtered data are sent to the PC. The system uses a DVS camera communication protocol to receive real events from a 128 × 128 camera, and the LDSI filtering algorithm with 126 × 126 layer size according to the structure described in previous sections.

The total logic resource occupation for the camera communication protocol, the LDSI algorithm, AER packet creation and PC transmission was 671 LUTs, which is an impressively low value. In part, the reduced logic occupation is due to internal block RAM use for parameter storage: 40 internal FPGA block RAM were used. No additional FPGA resources were used for computation. Concerning the speed of operation, we used the on board 50 MHz clock but the implementation results showed that the system could run up to 177 MHz, providing enough speed for real time camera event processing and AER output generation.

In addition, the low computational complexity of the proposed algorithm would allow the use of low cost hardware device, such as simple microprocessor or microcontrollers. In fact, we also tested the system using an ATMega microcontroller and results were satisfactory up to a certain number of events per second. In the case of the FPGA, it can be guaranteed that all possible events for a 128 × 128 camera can be processed in real time.

## 5. Discussion

Concerning the results obtained for the hardware implementation, using the same LDSI parameters, the same scene was compared between results generated by the FPGA and the jAER software under PC, providing the same results in the FPGA and PC software, which validates the correct FPGA implementation.

**Figure 12:**
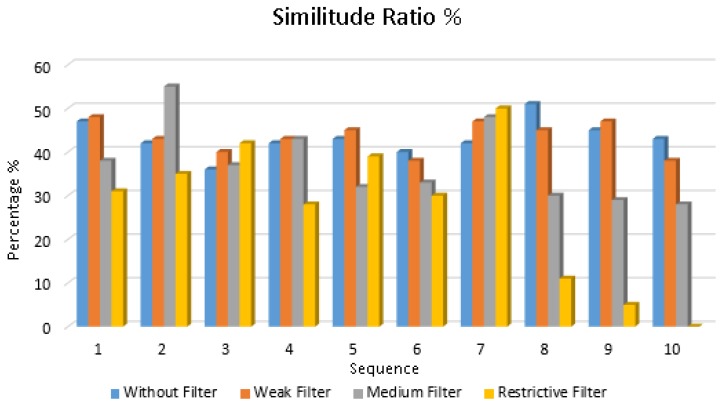
Similitude algorithm results for unfiltered input event data and three LDSI configurations: weak, medium and restrictive. As different scenes appear over time, the similitude ratio slightly changes. Low and medium LDSI configurations provide similar results to the original, at a higher data reduction.

Additionally, to verify the LDSI behaviour, once the LDSI algorithm has proven its capability for reduction of output data from an event-based scene, it is also necessary to verify that the relevant information in the scene is retained by the LDSI output data. Despite it can be clearly observed visually using jAER, a test pilot was conducted to formally verify this assumption. In this test, the original event data generated by a DVS camera and the event data produced by the LDSI algorithm after processing the same events were converted into a sequence of images generating a frame every 100 ms. A standard similitude algorithm typically used in industrial machine vision was applied, the algorithm provided the percentage of similitude between the LDSI output image values and a pattern initially shown to the algorithm as the master pattern. Thus, the original input data and all different LDSI configurations resulted in a similitude value compared to the initial pattern. The similitude value provides a comparison between the initial pattern and the LDSI configuration; the aim in this case was not to reach a high percentage value rather than a comparison among them. Figure 12 shows the result of the similitude algorithm applied to nine consecutive time sequences (100 ms each) for the same scene. This algorithm was based on the Mean Structural SIMilarity (MSSIM) index [36]. The similitude algorithm was evaluated for the original unfiltered data and three LDSI parameter configurations corresponding to those shown in Table 1 (weak, medium and restrictive). As seen, high data reduction was obtained while similitude results did not greatly differ from all four cases. However, higher similitude values in medium and weak LDSI parameterization with high data reduction were found when compared with the original unfiltered data (Figure 12). This fact shows that not only data reduction and denoising was obtained, but also the quality of image was increased when the LDSI filter was applied, e.g., edges were better defined.

Table 2 shows the data reduction values for the similitude analysis. As expected, the similitude ratio decreases with higher data reduction. Beyond 85% data reduction, the recognition was unsatisfactory with a high variation from one case to another (high standard deviation). However, data reduction up to 33% (weak filter) can be achieved while maintaining similar recognition results as the original data. Even in the case of 66% data reduction, the recognition ratio only drops 6.6% compared to original data. In summary, we can state that a high data reduction can be achieved in the case of weak and medium LDSI filter configurations (33% and 66% data reduction compared to the original, respectively), while maintaining a high similitude ratio.

**Figure 13:**
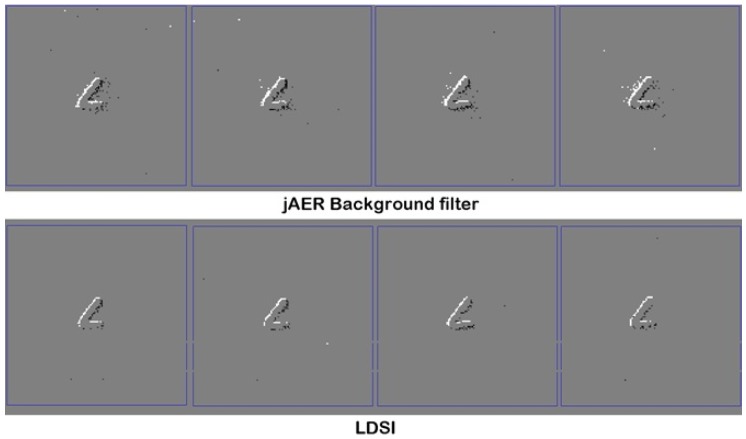
Filtering result for the background filter algorithm included in jAER software (**top**) and LDSI result (**bottom**).

A second test was conducted by comparing the LDSI filter result with other filtering algorithms. In this case, we used the jAER background filter algorithm. Tuned to provide the best performance, Figure 13 shows how the background filter does not remove all spurious events. The LDSI filter provides fewer spurious events and makes the relevant scene more defined.

**Figure 14:**
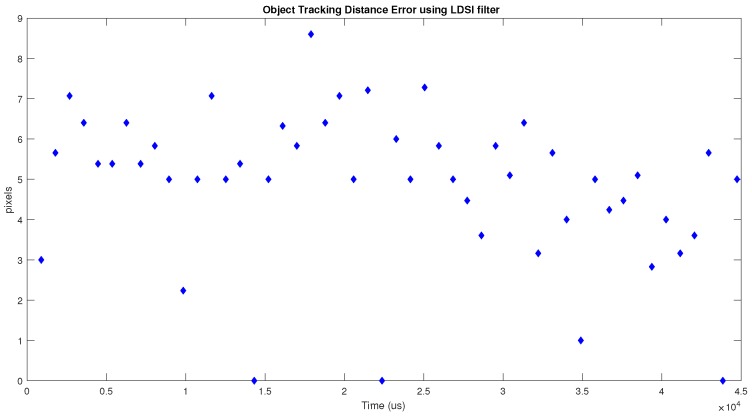
Error values in object tracking (actual position vs. tracking algorithm position) when LDSI filter is used.

**Figure 15:**
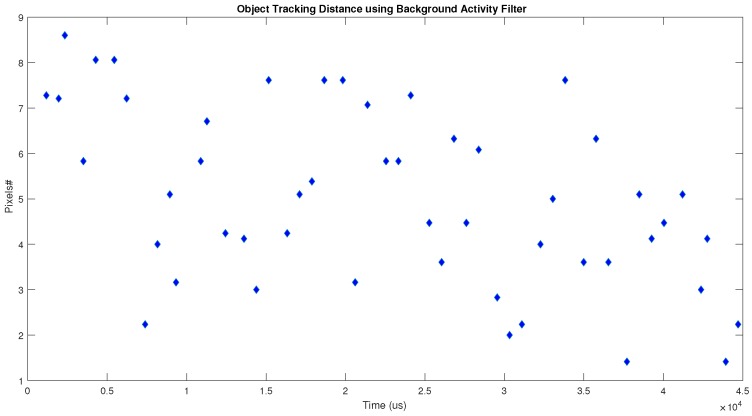
Error values in object tracking (actual position vs. tracking algorithm position) when the jAER built-in background activity filter is used.

To provide numerical results in the comparison between the LDSI and background activity filters, we applied the jAER built-in tracking algorithm, which provides the position of a moving object. For each filter, we obtained the error distance between the actual object position and the position given by the tracking algorithm. Figure 14 shows the results provided by the LDSI algorithm. Figure 15 shows the results obtained by the background activity filter. The error distance was calculated as the Euclidean distance in number of pixels. The obtained values were 4.86 ± 1.87 for LDSI, and 5.01 ± 1.93 for the background activity filters.

Figure 14 shows a more regular behaviour along time and, in some cases, the error is zero. However, Figure 15 shows that the background activity filter provides a more irregular behaviour, being affected by those events not removed from the scene. Since the LDSI removes more noisy events, it allows the tracking algorithm to provide more accurate and regular results.

## 6. Conclusions

A bioinspired filter for event-based systems is proposed, based on layers of units inspired in biological neurons and their interconnections. We can conclude that the initial aim was achieved, i.e., a configurable filter providing a range of options in the result, from transparent to zero output event mode. It was demonstrated that reducing the amount of data and noise with different levels depends not only on the parameter values, but also on the interaction among them and the types of input data (fast scenes, high intensity level change, etc.). For that reason, the LDSI algorithm can be configured to be adapted to different situations due to its parameters.

Filtering algorithms for event-based cameras are seldom reported, and they are designed for specific applications. The LDSI filter is compared to a commonly used jAER software filter, providing improved results, especially in complex situations with fast moving objects in the scene. The LDSI filter can be used in any scenario where the input consists on a matrix sensor generating events, not only image sensors but also other event sensors, such as auditory [3], distance measurement or magnetic compass [34], olfactory systems [4,5], and tactile [37] sensors.

The filter was tested with a DVS camera showing that noise reduction is improved, with a reduction in produced data, too. Produced data can reach 30% event data reduction compared with the original event-based generated image with an improvement in the scene definition and noise reduction. Usually, event-based cameras produce fewer data than conventional frame-based cameras and the proposed algorithm obtains a higher reduction. This reduction ratio becomes very important when data have to be transmitted from the camera to other processing systems as neuromorphic devices or Spiking Neural Networks (SNN), especially in cases where the same communication channel has to be shared among multiple devices, e.g., an ethernet-based communication. In addition to better scene definition, the LDSI allows a reduced AER data flow, which can reduce data congestion in communication channels.

Even though multiple combinations of parameters are possible, an analysis is done to provide a guideline on the parameter configurations leading to different output filter events. The filter is fully configurable; in this work, three parameter sets were proposed: low data reduction keeping all image features (weak filter), medium data reduction with some possible loss of information (medium filter) and strong data reduction with some loss of information (restrictive filter). We recommend watching the videos included as Supplementary Materials to evaluate the performance of LDSI filter.

Finally, LDSI hardware implementation shows that low resource usage is required, being an option for event-based sensor processing as on-board filtering before transmitting data to other devices or feeding data to neuromorphic system. This is possible due to the specific LDSI design having in mind that it can be used as an intermediate processing block, fully compatible with AER input data from an event-based sensor and generating AER output data as if data were generated by the sensor, thus being “transparent” to devices receiving data. Furthermore, its simplicity would allow its implementation in low cost microcontrollers.

## Figures and Tables

**Table 1:** Parameter values for different LDSI filter configuration and the associated number of events generated for the image shown in Figure 11. The original number of events from source with no filtering is also shown.

Parameter	Original	Weak	Medium	Restrictive
MTR	-	400	400	400
ERCO	-	2	5	1
TCE	-	3	5	2
TNE	-	2	6	8
ERCN	-	2	4	4
DERP	-	3	1	1
ERNC	-	3	4	4
DERC	-	3	1	1
Total events	25,596	17,196	8616	3769

**Table 2:** Data reduction and similitude test for original DVS camera data (unfiltered), and different LDSI parameters.

	Size (bytes)	Reduction (%)	Simil. Ratio (%)
Original data	153,576	-	43.02 ± 4.01
Weak filter	103,176	32.82	43.04 ± 3.68
Medium filter	51,696	66.34	37.64 ± 8.92
Restrictive filter	22,614	85.28	27.5 ± 16.52

## Supplementary Materials

Different video recordings were created to appreciate the filter performance under different filter parameters and scenes: Raw data from event-based camera without filtering https://youtu.be/ogpwNKAEWF0. LDSI algorithm output with a weak level of filtering https://youtu.be/ynNhRVe_SG4. LDSI algorithm output with a medium level of filtering https://youtu.be/XQbcV-ANoh0. LDSI algorithm output with a high level of filtering https://youtu.be/fmL8TPMGNZw. jAER tracking on event data with jAER background filter: https://youtu.be/7nptQ9EMWc8. jAER tracking with LDSI: https://youtu.be/YgIgVSa5JNY. Tracking algorithm results for low, medium and restrictive filter parameters in the case of the LDSI and background activity filters: https://www.youtube.com/playlist?list=PLgZjGCa1fp1EhRM6ctrWxv3jTyfnndXRJ.
